# Abdominal Visceral Injury, a Devastating Consequence of Abdominal Liposuction: A Case Report and Literature Review

**DOI:** 10.7759/cureus.34378

**Published:** 2023-01-30

**Authors:** Mohamed H Khalaf, Mohammed Sameer, Mohammad B Khan, Aryan Ahmed

**Affiliations:** 1 General Surgery, Hamad Medical Corporation, Doha, QAT; 2 Acute Care Surgery, Hamad Medical Corporation, Doha, QAT

**Keywords:** liposuction complications, visceral injury, acute care surgery, bowel perforation, liposuction

## Abstract

Abdominal liposuction is a commonly performed cosmetic procedure. However, as with any procedure, it can be associated with complications. One of the life-threatening complications of this procedure is visceral injury and bowel perforation. This complication is very rare, nevertheless general, and acute care surgeons must be aware of its possibility, its management, and its possible sequelae. We report a case of a 37-year-old female who underwent abdominal liposuction which was complicated by bowel perforation and was transferred to our facility for further care. She underwent an exploratory laparotomy in which multiple perforations were repaired. The patient then underwent multiple surgeries including stoma creation and had a long postoperative course. A literature review reveals the devastating sequelae of reported similar visceral and bowel injuries. The patient eventually did well and her stoma was reversed. This patient population will require close intensive care unit observation and a low threshold of suspicion for missed injuries during initial exploration. Further down the line, they will need psychosocial support and the mental health implications of this outcome must be cared for. The long-term aesthetic outcome is yet to be addressed.

## Introduction

Abdominal liposuction is one of the most common and routinely performed cosmetic procedures, however, it can have complications. The procedure can be performed in the outpatient setting making it appealing to the public and possibly downplays the potentially deadly complications that may occur. Complication rates are reported between 0% to 10% compared to mortality rates reported at <1% at the hands of experienced plastic surgeons. Bowel and visceral perforation during liposuction are rare complications with a reported incidence of <0.1 [1,2]. In 1989, an initial report of peritoneal perforation during liposuction was published, where two patients were explored due to this complication [3]. Major complications resulting in mortality after liposuction include pulmonary emboli followed by bowel perforation [2].

Some reports have documented the detrimental effect of this complication leading to death in several reported cases [4]. Many of these patients present with peritonitis, shock, and extensive necrotizing fasciitis, and despite best efforts, cannot be saved [5,6]. We present a patient referred to our hospital two days after liposuction resulting in small bowel injury with a complicated post-operative course. We aim to shed light on a potentially devastating complication of a routine procedure and how it was managed.

## Case presentation

A 37-year-old female with no past medical history and a past surgical history of abdominoplasty and umbilical hernia repair, with a body mass index of 28, was transferred from an outside hospital to our emergency department two days after undergoing abdominal liposuction. She had developed generalized abdominal pain after the procedure, which was worsening in severity, and unresponsive to analgesics.

On arrival at our institution, the patient was in evident distress and pain, she had a heart rate of 160 beats per minute, a respiratory rate of 43 breaths per minute, blood pressure of 105/68 mm/Hg. Abdominal examination revealed distension with generalized tenderness, guarding all over the abdomen, and visible well-healed scars from her prior abdominoplasty. Labs were significant for a white blood cell count of 12.5 x 103/uL, hemoglobin of 15.4 gm/dL, urea of 7.8 mmol/L, creatinine of 82 umol/L, and a lactate of 4.2 mmol/L.

**Figure 1:**
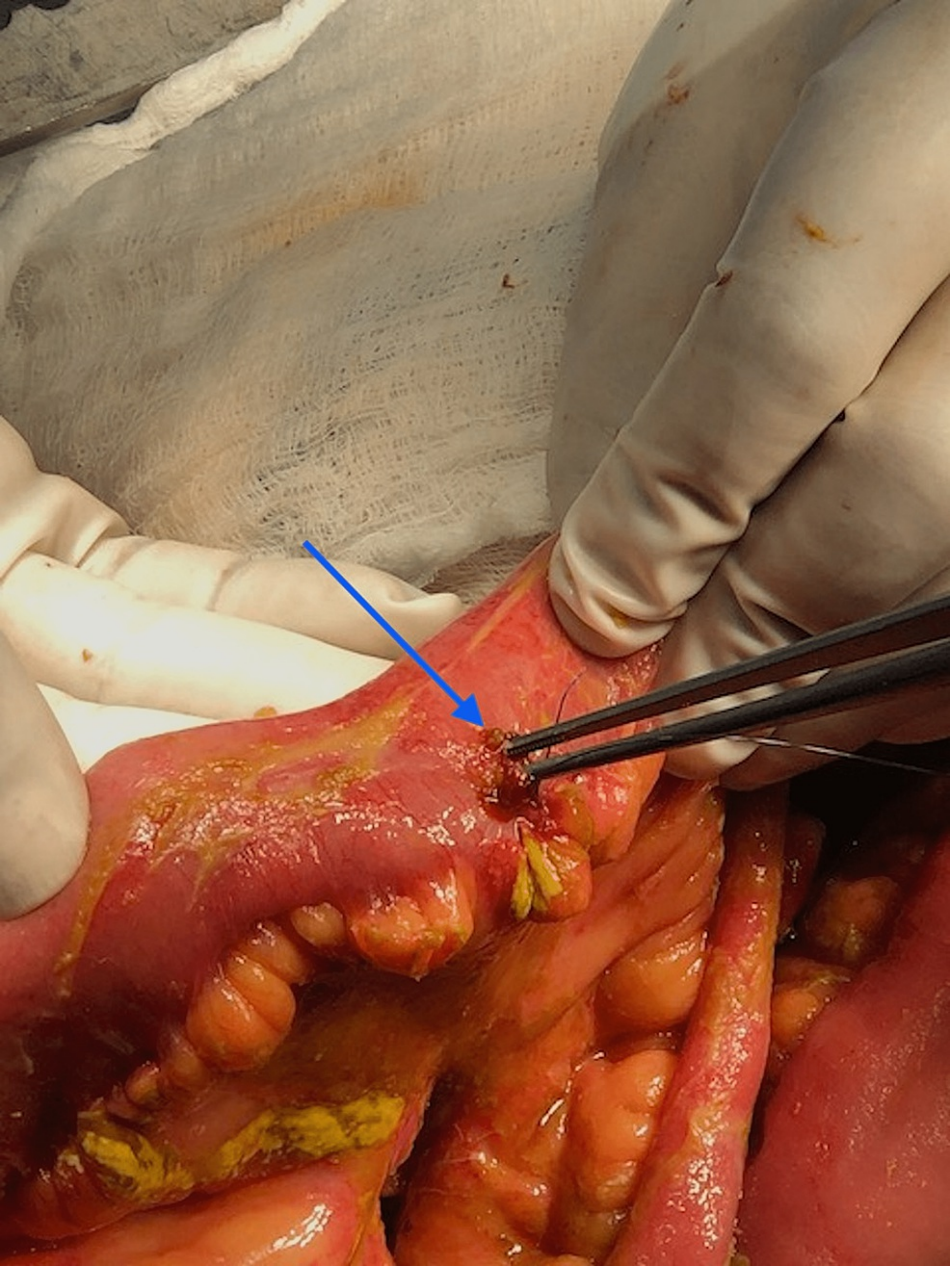
One of the small bowel perforations identified during the initial surgery

**Figure 2:**
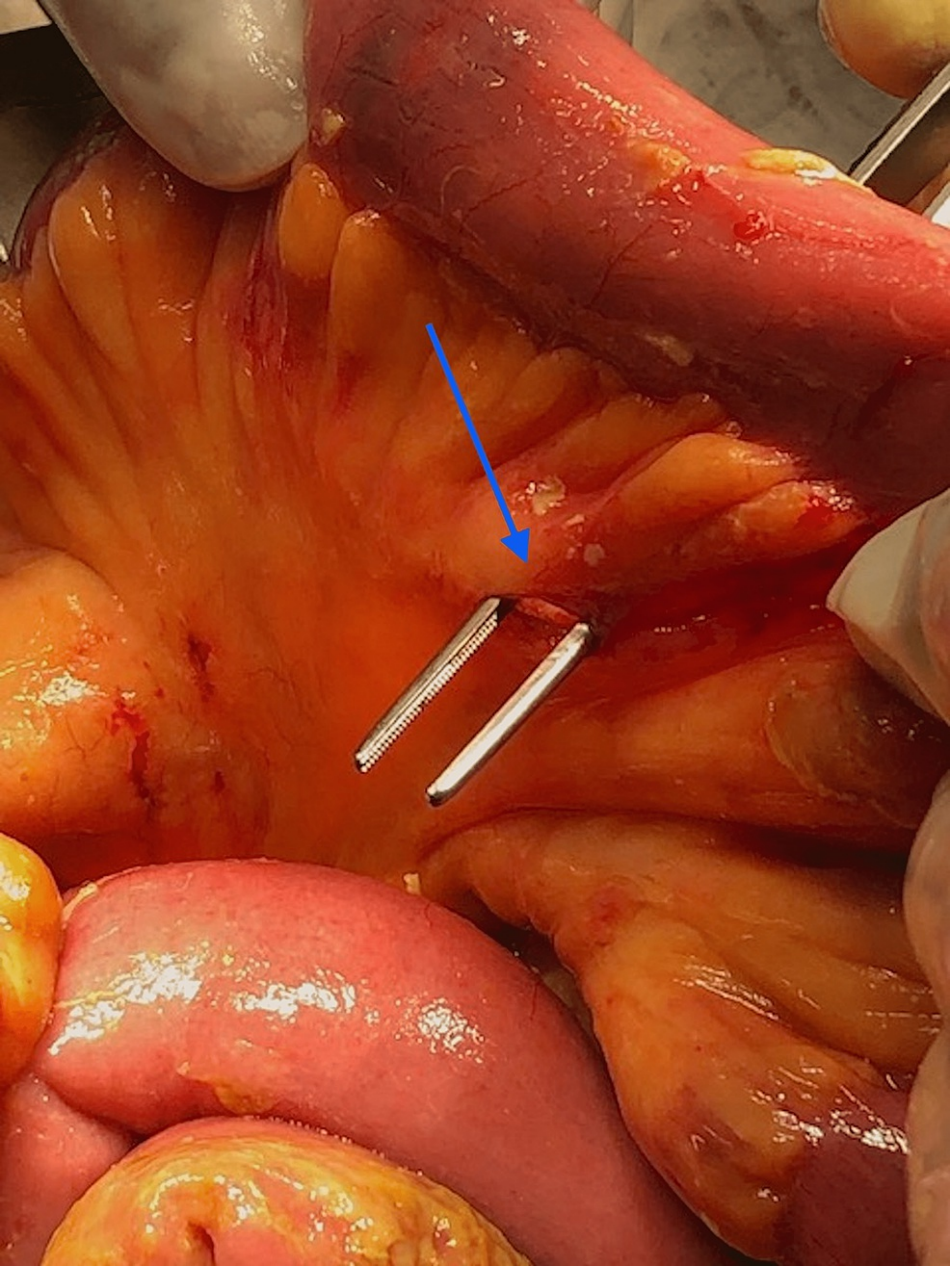
Mesenteric laceration encountered on initial exploration

**Figure 3:**
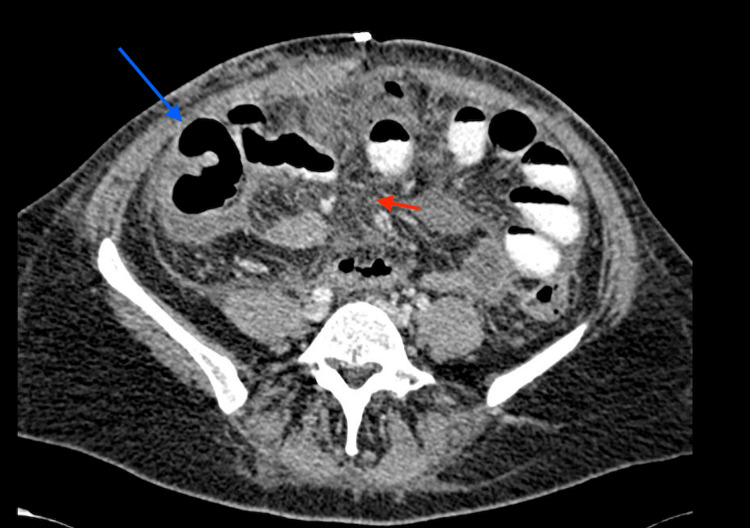
Repeat CT scan after initial surgery showing dilated bowel loops (blue arrow) and mesenteric fat stranding (red arrow). No evidence of a contrast leak.

A CT scan had already been done at the referring facility. We were able to review the images at the time which showed free air in the peritoneal cavity and a significant amount of free fluid. She was diagnosed with perforated viscous post-abdominal liposuction. The patient was stabilized with intravenous fluids and antibiotics and prepared for emergency surgery. The patient was taken for an emergency exploratory laparotomy which revealed four enterotomies (Figure 1), mesenteric laceration (Figure 2), and five seromuscular injuries in the small bowel. All bowel injuries were repaired primarily, and the abdomen was closed. Her condition did not improve in the following days, and a repeat CT scan showed mesenteric fat stranding, dilated bowel loops and no evidence of contrast leak (Figure 3).

**Figure 4:**
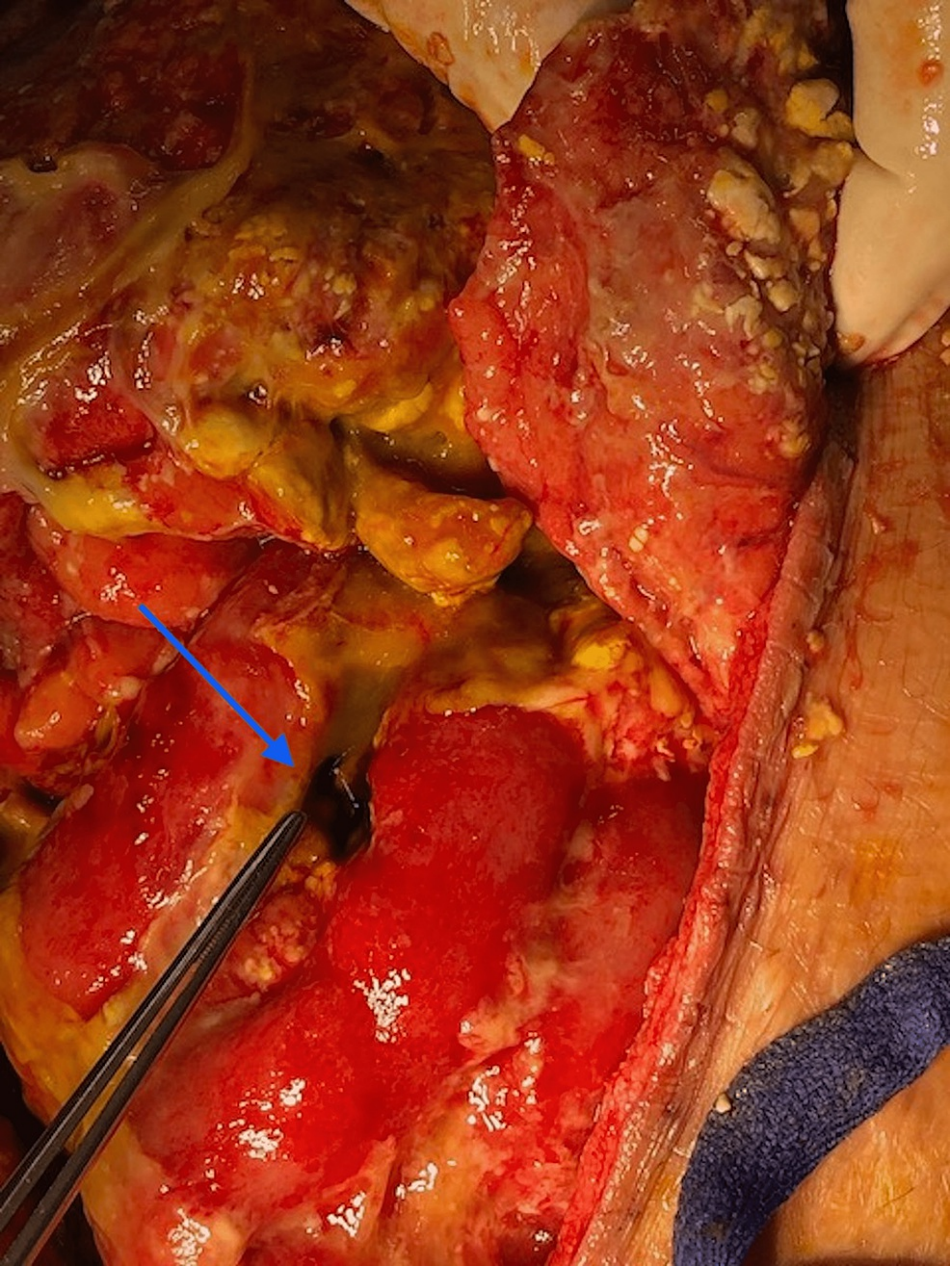
One of the newly discovered enterotomies on re-exploration

She had rising inflammatory markers, interval development of bacteremia, pleural effusion, and a persistent fever. The patient was re-explored on postoperative day 5, where two further enterotomies (Figure 4) were identified including one on the mesenteric border of the small bowel in addition to persistent leak from prior repair sites. The affected segment with the persistent leak was resected and bowel continuity was restored, the abdomen was kept open.

A relook surgery on postoperative day 8 revealed persistent intestinal content leak from the previously repaired perforations and additional mesenteric border perforation was identified, and the patient underwent further bowel resection. During the surgery, the patient was hemodynamically unstable with an increasing requirement for vasopressor support. We opted not to anastomose at the time and she was left with an open abdomen for planned re-exploration. On postoperative day 10, she was taken back for relook surgery where a peri splenic abscess was drained and additional perforation was identified and resected, with the creation of a double barrel ileostomy and application of vacuum-assisted closure (VAC) device for her midline wound. Her abdominal wound was eventually partially approximated with the aid of a bridging mesh on postoperative day 13.

She had a turbulent postoperative course with ongoing bacteremia, candidemia, and altered mental status requiring reintubation. She was supported with total parenteral nutrition and transitioned to nasogastric feeds until she tolerated the oral diet. She eventually recovered with the aid of physical and speech therapy. She was discharged two months after admission.

The patient was referred to the plastic surgery service. She underwent partial thickness skin grafting of her abdominal wound with a favorable outcome. In the interim, she has had multiple visits to the emergency with fatigue, vague abdominal symptoms, and signs of dehydration requiring fluid resuscitation due to high stoma output. She eventually underwent a successful reversal of her stoma and the repair of an incisional hernia.

## Discussion

Liposuction is a common procedure and its popularity is on the rise [7,8]. Our case report highlights a potentially fatal complication of a routinely performed procedure. Emergency medicine, plastic, and acute care surgeons must be aware of the potential complications associated with this seemingly safe, straightforward procedure. The commonly encountered complications after liposuction are usually mild and can be managed conservatively. However, some life-threatening complications must be kept in mind [9-11]. A review of the literature on similar cases identified 45 cases where patients had undergone abdominal liposuction complicated by visceral injury. Table 1 summarizes presenting demographics, symptoms, signs, and history.

**Table 1:** Summary of reviewed literature featuring baseline patient demographics, presenting signs and symptoms, procedures performed, and past medical and surgical history. AL: Abdominal liposuction, NR: Not reported, AP: Abdominoplasty, TRAM: Transverse rectus abdominis myocutaneous

Case	Author	Age	Sex	Symptoms	Sign	Procedure	History
1	Coronado-Malagon et al. [12]	54	F	Pain, nausea, malaise	Abdominal tenderness	AL	Spine surgery
2	Cuenca-Pardo et al. [13]	43	F	Pain, distension, dyspnea	Tenderness, distension, absent bowel sounds, guarding	AL	Appendectomy, CS, liposuction
3	De la Fuente et al. [14]	44	F	Pain, fever, nausea and vomiting	Hypotension, fever, tachypnea, tachycardia	AL	Sleeve gastrectomy, hypertension
4	Delliere et al. [15]	56	F	Pain	Shock, necrotizing cutaneous lesions	AL	-
5	Di Candia et al. [16]	52	F	Distension, vomiting, constipation	Septic shock	AL	-
6	Ezzeddine et al. [17]	26	F	Dyspnea, fatigue, pain	Tachypnea, pleural effusion	AL	None
7	Gardner et al. [18]	69	F	Vomiting	Tachycardia, desaturation	AL, AP	Open kidney surgery
8	Giordano et al. [19]	70	F	Pain, nausea, vomiting, dyspnea	Tenderness, distension	AL, mastopexy	None
9	Lehnhardt et al. [9]	NR	NR	-	-	AL	-
10	Lehnhardt et al. [9]	NR	NR	-	-	AL	-
11	Lehnhardt et al. [9]	NR	NR	-	-	AL	-
12	Lehnhardt et al. [9]	NR	NR	-	-	AL	-
13	Lehnhardt et al. [9]	NR	NR	-	-	AL	-
14	Lehnhardt et al. [9]	NR	NR	-	-	AL	-
15	Lehnhardt et al. [9]	NR	NR	-	-	AL	-
16	Lehnhardt et al. [9]	NR	NR	-	-	AL	-
17	Lehnhardt et al. [9]	NR	NR	-	-	AL	-
18	Mallappa et al. [20]	63	M	Pedal edema, dyspnea, fever, abdominal pain and distension	Fever, tachycardia, tachypnea, desaturation, ecchymoses	AL, umbilical hernia repair	-
19	Marques Álvarez et al. [21]	32	M	Pain, vomiting, fever	Hemodynamic instability, respiratory failure, distention, bloody wound discharge	AL	-
20	Ovrebø et al. [22]	56	F	Pain	Peritonitis	AL	Laparotomy, abdominoplasty, liposuction
21	Pohlan et al. [23]	29	F	Pain	Free fluid on ultrasound	AL	Dyslipidemia
22	Raman et al. [24]	56	M	Discharge from the abdominal wound	Colocutaneous fistula	AL	Abdominoplasty
23	Reddy et al. [25]	56	M	Pain and bloating	Abdominal wall erythema and crepitus, fever, tachycardia	AL	-
24	Sharma et al. [6]	55	F	Pain, altered mental status, wound discharge, bruising	Shock, necrotizing cutaneous lesions	AL, mastopexy	-
25	Singh et al. [26]	65	M	Pain, distension, obstipation, discoloration of the skin	Tachycardia, tachypnea, distension, subcutaneous emphysema	AL	Gastric bypass
26	Taha et al. [27]	41	M	Distention, pain, anorexia	Bilious drain output	AL	Laparotomy, sleeve gastrectomy
27	Talmor et al. [4]	50	F	Fever	Shock, distended abdomen	AL, rhytidectomy	-
28	Zakine et al. [5]	55	F	Pain, nausea, vomiting	-	AL, AP	Diastasis
29	Zakine et al. [5]	72	F	Pain	Shock	AL	Abdominal surgery, abdominoplasty, diastasis,
30	Zakine et al. [5]	45	F	Vomiting	Shock, obstruction	AL, AP	Abdominal surgery, diastasis, cesarian section
31	Zakine et al. [5]	44	F	Pain, nausea, vomiting	Guarding, obstruction	AL	-
32	Zakine et al. [5]	24	M	Pain, fever, dyspnea	Edema	AL	Abdominal surgery
33	Zakine et al. [5]	43	F	-	Hypovolemic shock	AL	Abdominal surgery, appendectomy
34	Zakine et al. [5]	50	F	Pain	Guarding, shock	AL	Abdominal surgery, nephrectomy, hysterectomy
35	Zakine et al. [5]	57	F	Pain	Guarding	AL, AP	Abdominal surgery, diastasis, liposuction
36	Zakine et al. [5]	66	F	Pain, nausea	-	AL	Abdominal surgery, abdominoplasty
37	Zakine et al. [5]	44	F	Pain	Guarding	AL	-
38	Zakine et al. [5]	62	F	Pain, nausea, vomiting	-	AL	Abdominal surgery, TRAM flap
39	Zakine et al. [5]	53	F	-	Hemodynamic shock	AL, AP	Abdominal surgery, diastasis, appendectomy, inguinal hernia
40	Zakine et al. [5]	47	M	Pain	Guarding	AL	-
41	Zakine et al. [5]	52	F	Pain	Guarding	AL	Abdominal surgery, umbilical hernia
42	Zakine et al [5]	40	M	Pain	Guarding	AL	Diastasis
43	Zakine et al. [5]	43	F	Pain, vomiting	-	AL, AP	Umbilical hernia, CS, tubal ligation
44	Zakine et al. [5]	53	F	Pain	Guarding	AL	Abdominal surgery, cholecystectomy, sigmoidectomy, umbilic hernia
45	Zakine et al. [5]	62	F	Pain	Guarding	AL	Abdominal surgery, hysterectomy, cholecystectomy
46	Zakine et al. [5]	54	F	Pain	Guarding	AL	Abdominal surgery, appendectomy, diastasis

Of the 45 patients outlined, 36 patients had past surgical history reported. It’s evident that 20/36 (55%) of patients had prior abdominal surgery. Zakine et al. identified and outlined abdominal diastasis in seven of the nineteen (36%) reported patients with bowel perforation [5]. Table 2 shows the imaging modality and findings, management, and outcomes. Unfortunately, of the reported cases, seven deaths (16%) were reported. A review of the outcomes shows intensive care unit stays, prolonged hospital stays, and the need for multiple surgeries. Another associated problem is necrotizing fasciitis requiring extensive debridement. Some patients present within a day others presented after a week. Injuries may involve the liver, gall bladder, spleen, and small and large bowel.

**Table 2:** Summary of literature review imaging findings, management, affected organs, and outcomes D/c: Discharge, ant.: Anterior, POD: Postoperative day, wks: Weeks, D and d: Day, NR: Not reported, TPN: Total parenteral nutrition, ICU: Intensive care unit, EC: Enterocutaneous, MRCP: Magnetic resonance cholangiopancreatography

Case	Imaging	Imaging finding	Management	Outcome	Mortality	Time to symptoms (days)	Location	Number of perforations
1	CT	Free air in the abdomen	Laparotomy, resection anastomosis	Unremarkable	-	1	Jejunum	Single
2	XR	Air under diaphragm	Laparotomy, resection anastomosis	Re-exploration for obstruction on POD7	-	2	Jejunum	-
3	CT	Fluid collection	Laparotomy, necrotizing fasciitis, mesenteric abscess, small bowel perf	Multiple surgeries, 31 days in the hospital	-	8	Small bowel	Multiple
4	CT	Free air, bilateral congenital lumbar hernia	Releasing incisions, laparotomy	12-week hospital stay, colostomy	-	1	Descending colon	Multiple
5	None	-	Laparotomy, partial bowel resection, multiple relooks and resections	ICU 3 weeks, end jejunostomy and colostomy reversed after 10 months. Component separation	-	4	Small and large bowel	Multiple
6	CT, MRCP	Liver tracts extending to the pleura, pericholecystic fluid, empty gall bladder	Laparoscopic exploration, cholecystectomy	Discharged POD2	-	5	Liver, gall bladder	-
7	CT	Free air, free fluid	Resection anastomosis	Unremarkable	-	2	Bowel	Multiple
8	CT	Intra-abdominal fluid, distended bowel	Laparotomy, primary repair	Discharged POD7	-	7	ileum	-
9	-	-	-	Death	Y	-	Small intestine	-
10	-	-	-	Death	Y	-	Small intestine	-
11	-	-	-	Death	Y	-	Small intestine	-
12	-	-	-	NR	-	-	Small intestine	-
13	-	-	-	NR	-	-	Small intestine	-
14	-	-	-	NR	-	-	Small intestine	-
15	-	-	-	NR	-	-	Small intestine	-
16	-	-	-	NR	-	-	Colon	-
17	-	-	-	NR	-	-	Gall bladder	-
18	XR	Air under diaphragm	Laparotomy, primary repair	Cardiac arrest, ICU 7 days, ICU psychosis	-	6	Ileum	Multiple
19	CT	Subcutaneous emphysema, pneumomediastinum, pneumoperitoneum, free fluid in the abdomen	Laparotomy	ICU, decompressive laparotomy, MODS, death POD3	Y	3	Ileum	Multiple
20	XR	Free air	Laparotomy, resection anastomosis	Unremarkable	-	<12 hours	Intestine	Multiple
21	CT	Liver hematoma	Supportive care	Unremarkable	-	<12 hours	Liver	Multiple
22	CT	Subcutaneous emphysema	Drainage of anterior abdominal wall collection, TPN, conservative	Spontaneous closure	-	3	Colon	Single
23	None	-	Laparotomy, debridement, resection	ICU, relook, EC fistula, laparotomy, and fistula resection 6 months later	-	6	Small bowel	Multiple
24	CT	Free air, ruptured implant	Laparotomy, skin releasing incisions, resection anastomosis	Death	Y	1	Ileum	Multiple
25	CT	Bowel obstruction	Primary repair of perforation	Sepsis and incisional hernia	-	7	Bowel	Single
26	CT	Bowel perforation	Resection anastomosis	Unremarkable	-	2	Bowel	Single
27	CT	Free air, dilated bowel loops	Laparotomy, resection anastomosis	Intra-abdominal collection, d/c day 25	-	1	Small bowel	Multiple
28	CT	-	Laparotomy, ileostomy	ICU 10 days, d/c day 20, hypertrophic scar	-	4	Ileum	-
29	CT	-	Laparotomy, hemicolectomy	Mesenteric infarction, death POD2	Y	2	TC, ileum	-
30	CT	-	Laparotomy, ileostomy	ICU 14 days, drainage of pelvic and pulmonary abscess, d/c day 17	-	7	Ileum	-
31	XR	-	Laparotomy, jejunostomy	ICU 16 days, d/c day 28	-	3	Jejunum	-
32	CT	-	Laparotomy, ileostomy	ICU 7 days, d/c day 17, posttraumatic neurosis	-	6	Ileum	-
33	CT	-	Laparotomy, splenectomy	ICU 10 days, thrombophlebitis, pleural collection, d/c day 17	-	1	Spleen	-
34	CT	-	Laparotomy, ileostomy	ICU 21 days, d/c day 40, chronic pain, depression, hernia	-	1	Ileum	-
35	XR	-	Laparotomy, ileostomy	ICU 19 days, d/c day 42, chronic abdominal pain, depression	-	5	Sigmoid colon, ileum	-
36	CT	-	Laparotomy, resection anastomosis	ICU 57 days, pleural collection, D/c 6 months, post-traumatic neurosis, depression, pain, hernia	-	5	Ileum	-
37	None	-	Laparotomy, primary repair and re-exploration	Septic shock, death POD11	Y	6	Cecum	-
38	CT	-	Laparotomy, ileostomy	ICU 16 days, d/c day 37	-	2	Jejunum	-
39	CT	-	Laparotomy, splenectomy	ICU 3 days, d/c day 14, post-traumatic neurosis, hernia	-	0	Spleen	-
40	CT	-	Laparotomy, ileostomy	ICU 14 days, d/c day 14, pleural collection, abdominal hernia	-	5	Ileum	-
41	CT	-	Laparotomy, ileostomy	Septic shock, death POD5	Y	4	Ileum	-
42	CT	-	Laparotomy, primary repair	ICU 10 days, d/c day 19, pain	-	5	Ileum	-
43	CT	-	Laparotomy, ileostomy	ICU 45 days, d/c 5 months, pain, hernia	-	3	Ileum	-
44	CT	-	Laparotomy, primary repair	Pain	-	1	Ileum	-
45	CT	-	Laparotomy, primary repair	ICU 8 days, d/c day 18 hernia	-	1	Ileum	-
46	CT	-	Laparotomy, ileostomy	ICU 15 days, d/c 2 months	-	3	Ileum	-

Grazer (&) De Jong reported 95 deaths in a survey of 496,245 reported liposuction procedures. Perforation was the second leading cause after pulmonary embolism [28]. As evident from our review, these patients present in a critical state with active sepsis and shock and require emergency surgery. With our patient, she had multiple missed perforations which declared themselves on re-exploration. Given our experience with this patient, we would recommend planned relooks after initial exploration and meticulous examination of the bowel including the mesenteric border as well for any suspicious areas of perforation. It is also worth noting that the perforations repaired primarily failed to heal most likely due to the poor underlying condition of sepsis, bacteremia, and hostile abdomen with peritonitis. Further attention must be paid to nutritionally supporting this patient population from the moment they present in anticipation of future surgery.

## Conclusions

Abdominal liposuction is a well-established and safe procedure; however, we must be aware of its potentially life-threatening complications and how to deal with them. Our case report outlines our experience with a patient who recently underwent abdominal liposuction and her prolonged postoperative course in and out of the hospital setting. In this case, we had a favorable outcome despite her instability in presentation and complicated postoperative course. However, there is a mental health and psychosocial implication on the patient that has not been studied in this case.

Patients who undergo liposuction complicated by perforation require a multidisciplinary approach of emergency medical care, intensivists, surgeons, psychologists, and therapists to aid their recovery.
